# Transition Path Flight Times and Nonadiabatic Electronic Transitions

**DOI:** 10.1021/acs.jpclett.2c01425

**Published:** 2022-07-25

**Authors:** Xin He, Baihua Wu, Tom Rivlin, Jian Liu, Eli Pollak

**Affiliations:** † Beijing National Laboratory for Molecular Sciences, Institute of Theoretical and Computational Chemistry, College of Chemistry and Molecular Engineering, Peking University, Beijing 100871, China; ‡ Chemical and Biological Physics Department, Weizmann Institute of Science, 76100 Rehovot, Israel

## Abstract

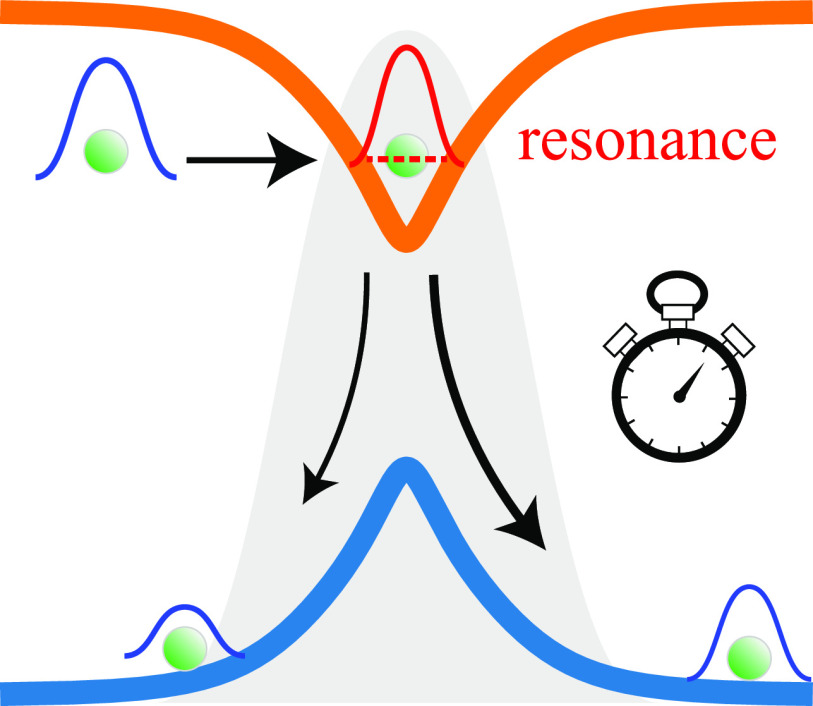

Transition path flight times are studied for scattering on two electronic surfaces with a single crossing. These flight times reveal nontrivial quantum effects such as resonance lifetimes and nonclassical passage times and reveal that nonadiabatic effects often increase flight times. The flight times are computed using numerically exact time propagation and compared with results obtained from the Fewest Switches Surface Hopping (FSSH) method. Comparison of the two methods shows that the FSSH method is reliable for transition path times only when the scattering is classically allowed on the relevant adiabatic surfaces. However, where quantum effects such as tunneling and resonances dominate, the FSSH method is not adequate to accurately predict the correct times and transition probabilities. These results highlight limitations in methods which do not account for quantum interference effects, and suggest that measuring flight times is important for obtaining insights from the time-domain into quantum effects in nonadiabatic scattering.

Questions surrounding quantum transition times such as tunneling durations,^1−6^ electronic transition times,^7−9^ and other interaction times^10,11^ have gained increased salience in recent years, as theoretical^12−17^ and experimental^18−25^ advances have encouraged researchers to look at conceptual issues surrounding them anew. In this context, we have shown previously that a weak-value-based mean time,^26,27^ called the transition path flight time, is a useful way to probe the time domain in the study of quantum transitions, notably in measuring transition times and demonstrating their connection to phase times in the context of tunneling.^16,28,29^ Here, we perform similar analysis to calculate the flight times associated with highly nonadiabatic transitions in a one-dimensional, two-level model system. Quantum effects such as tunneling, resonance and classically forbidden transitions are expected to play significant roles in determining these times in such systems.^30−34^ In particular, we calculate mean times and accompanying time distributions associated with the transmission and reflection subensembles on both of the two surfaces, alongside the associated transmission and reflection probabilities—studying these time-domain quantities reveals new physical insights that the probabilities alone fail to divulge.

To accurately calculate these quantities, we employ two numerically exact quantum propagation methods—the split-operator^35^ and the discrete variable representation (DVR)^36^ methods. The numerically exact quantum results are compared to the fewest switches surface hopping (FSSH) method originally developed by Tully,^37^ as well as its expanded versions.^38−42,65,66^ The initial condition of the nuclear degree of freedom in FSSH is treated quasi-classically in the present paper. FSSH is in wide use in the field of chemical dynamics,^42−53^ and it is considered to be a good method that semiquantitatively describes scattering in multidimensional systems with strong nonadiabatic coupling, despite the fact that the method cannot account for quantum effects such as tunneling and quantum interference. It is considered an improvement over the Ehrenfest dynamics (mean field) method.^51^ Surface hopping methods are, however, currently being reexamined in a variety of contexts,^53^ and so the question of whether the FSSH method produces accurate flight times for scattering is timely, yet also underexplored.

The nonadiabatic model system explored here is the same as the one originally proposed by Tully:^37^ a one-dimensional, two-level single-avoided crossing. We compare the numerically exact results with FSSH-generated results in different energy regimes—deep tunneling, above-threshold, and near resonances. We also explore how the width of the incident wave packet affects the dynamics and the final scattering observables. We find that, in many instances, quantum effects lengthen the flight time as compared with results obtained using FSSH or classical mechanics. The flight time distributions reveal resonance and threshold phenomena. Our FSSH results reproduce the same transmission and reflection probabilities as Tully did in his original work, yet a careful study of the resonance region reveals that the FSSH method misses here the characteristic oscillations in the reflection probabilities.

Quantum mechanically, the quantity of interest is —the two-component vector of wave functions ,  corresponding to the two levels, as functions of position and time. The transmission and reflection probabilities are obtained by considering the fluxes through points far to the left and right for the two surfaces over time, and so a numerical method must be used to propagate an initial state, , in time.

In all of our computations, the initial state is chosen to be a Gaussian whose entire amplitude is on the ground electronic surface, centered at a point, *x*_0_, far to the left of the interaction region (which is centered on *x* = 0), and with an initial momentum centered around *ℏk*_0_ (*ℏ* is set to 1 in all subsequent equations):
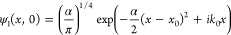
1where α is a width parameter. This state is related by a Fourier transform to
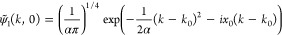
2

In principle there are four scattering channels: one reflection and one transmission channel for each of the two potential energy surfaces’ (PESs’) asymptotes (scattering channels are inaccessible below the asymptotic energy of the associated surface). Each channel will have an amplitude associated with it: *T*_1_, *T*_2_, *R*_1_, and *R*_2_. For time-independent scattering of energy eigenstates, these amplitudes are associated with the asymptotic wave functions of each of the four scattering channels. The magnitude squared of the amplitudes give the relevant probabilities as standard.

We calculate a mean time-of-flight for each of the four channels separately using a definition based on weak value theory:^16,28^
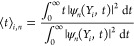
3where *n* = {1, 2} refers to the two levels and *Y*_*i*_ is a “screen” which is far to the left for reflected channels and far to the right for transmitted ones. Just as one can define quantities such as the “probability of transmission on the lower surface”, it is now possible to assign separate mean times to portions of the initial wave packet in each scattering channel.

The model studied here is the “simple avoided crossing” (SAC) model of Tully^37^ (shifted such that *E* = 0 is the lower asymptote). In the diabatic case, it has one crossing. The two diagonal components of the 2 × 2 diabatic potential energy matrix in the Hamiltonian are
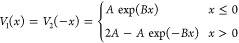
4

Hence *V*_1_ is the lower surface asymptotically to the left and the higher surface asymptotically to the right, and vice versa for *V*_2_. The off-diagonal components are
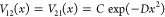
5In Tully’s work and here, the potential parameters in atomic units are *A* = 0.01, *B* = 1.6, *C* = 0.005, and *D* = 1.0, the particle mass *M* = 2000, and Planck’s constant *ℏ* = 1.

In the adiabatic representation, the two PESs are given by
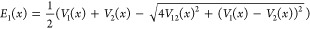
6
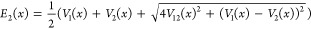
7and the nonadiabatic coupling strength is given by (primes denote derivatives)^54^
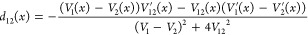
8

Adiabatically, the crossing is avoided, and the lower surface, *E*_1_, remains lower than the upper surface, *E*_2_, consistently. The potentials and couplings are shown in Figure 1. Despite its simplicity, the SAC model does have implications for realistic molecular systems.^55^

**Figure 1:**
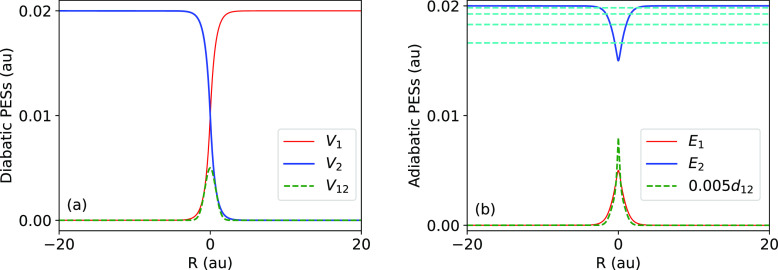
Tully’s “simple avoid crossing” (SAC) model, shifted upward. Panel a shows the diabatic surfaces, where red solid, blue solid, and green dashed lines denote *V*_1_, *V*_2_, and *V*_12_ (the off-diagonal term), respectively. Panel b shows the adiabatic surfaces *E*_1_ (solid, red) and *E*_2_ (solid, blue) and the nonadiabatic coupling term *d*_12_ (× 0.005, green dashed). The cyan dashed lines denote the lowest four bound energy levels in the upper adiabatic well computed by including the diagonal nonadiabatic coupling term.

Tully’s FSSH method uses swarms of trajectories propagated classically along PESs. The trajectories are assigned to scattering channels in the correct ratios by propagating a pair of wave function coefficients quantum mechanically. (These time-dependent coefficients should not be confused with the quartet of time-independent, asymptotic coefficients *T*_1_, *T*_2_, *R*_1_, and *R*_2_ introduced earlier.) The swarms are needed because it is a stochastic method: at each time step the wave function coefficients give probabilities of trajectories instantaneously “hopping” between surfaces (this is “frustrated” if there is insufficient energy). Hence many trajectories are needed to build the accurate statistics for the transmission and reflection probabilities.

To calculate flight time distributions, we simply count the number of time steps taken for the trajectory to cross the interaction region. More than 10^6^ trajectories are necessary to obtain sufficient statistics for the distributions. In this work, for the FSSH method, the initial momenta of the trajectories are sampled from a Gaussian momentum distribution to facilitate comparisons to quantum wave packet propagation results. (The specific sampling method has been known to significantly affect the final distributions in FSSH.^52^) The momenta are selected randomly from a distribution based on the magnitude squared of eq 2, which corresponds to the Wigner distribution of momenta.^16,53^ This demands an increase in the number of trajectories sampled.

It is well-known that the FSSH method accurately reproduces transmission and reflection probabilities in many systems. The method has been expanded upon many times over the years, most notably with adjustments to account for decoherence.^46,48^ In this letter, we use the well-known version of Tully’s algorithm.^37^ More expanded versions of surface hopping are tested in the Supporting Information. To date, (to the best of our knowledge) transition path flight times have not been studied using the FSSH-based methods.

We identify major differences in flight times and probabilities. First, at energies where tunneling effects on the lower surface are significant, second, at energies near resonances in the upper-surface adiabatic well, and third, when classically disallowed nonadiabatic transitions are significant. We calculated the eigenenergies of the upper surface well with the DVR approach with nonadiabatic corrections, and calculated mean times and probabilities around these energies (see Supporting Information, Section S1-B). There we found notable resonance effects in the quantum regime, but not with FSSH.

While the Tully method detected larger-than-expected flight times in the upper well region, since trajectories “hop” between surfaces and bounce around inside the well multiple times before leaving the interaction region, this did not compensate for the lack of resonance interactions, and so the flight times were always lower in this region for the FSSH method.

In all the numerical simulations, the center of the initial wave packet was *x*_0_ = −77.8617 atomic units and the screens were placed at *Y*_*i*_ = ± 145.723 (all further numbers will be in atomic units). A series of values in the range [0.006, 0.03] was used for the width parameter α, with α = 0.006 representing a narrow-in-momentum initial wave packet, and α = 0.03 a wide-in-momentum one. (See Supporting Information, Sections S1-D and S2-C, for all of the numerical parameters used, and Section S4, for more details on how the results varied with initial wave packet width.)

The mean scattering time and the scattering probability for all four channels were calculated via numerically exact quantum mechanical (QM) methods and by using FSSH. The two methods employed for the QM calculations—the discrete variable representation (DVR) and split-operator (S–O) methods (see Supporting Information, Sections S1-A and S1-B)—gave the same results within an acceptable accuracy of a few percent difference at most. To remove the trivial contribution to flight times due to motion in the asymptotic region and reveal the effect of the nonadiabatic dynamics on the fight times, the corresponding free-particle flight time *t*_fp_ from *x*_*i*_ to *Y* was subtracted from the mean scattering times.

Figure 2 shows the mean flight time difference as a function of initial kinetic energy (here the initial momentum *ℏk* is positive) for the three possible exit channels and the narrowest-in-momentum (α = 0.006) and broadest-in-momentum (α = 0.03) initial wave packets. Panels a and b of Figure 2 show the reflection times on the ground state surface, and panels c and d do the same for the transmission times. When the energy of the particle is lower than the adiabatic barrier height of the lower adiabatic surface, the reflection time on the lower surface obtained from FSSH agrees well with the quantum time. In this region, the quantum tunneling probability is small and reflection is the classically allowed process. FSSH is, however, not capable of providing the transition time for the transmitted part.

**Figure 2:**
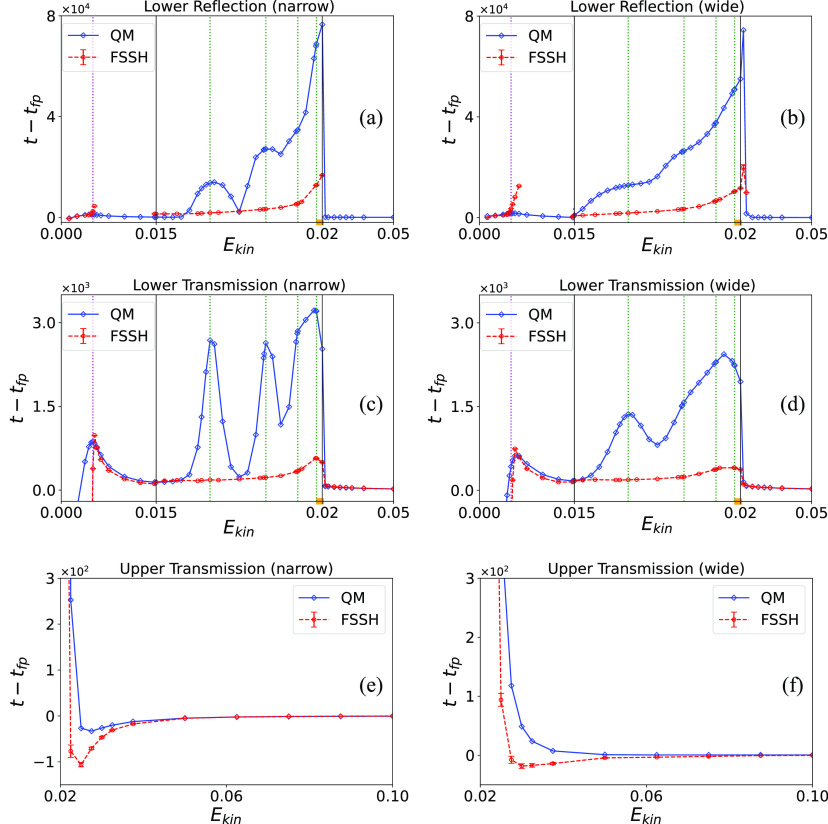
Mean flight time differences are plotted as functions of the initial mean kinetic energy. Panels a and b show mean flight times for the part of the distribution reflected on the lower surface with width parameters α = 0.006 and α = 0.03, respectively. Panels c and d are the same as panels a and b but for transmission probability on the lower surface. Panels e and f are the same but for transmission to the upper surface. For these latter two panels, the energy scale focuses on the above-threshold regime. In each panel, blue diamonds and red points represent results for the QM and FSSH methods respectively (blue solid and red dashed lines are only used to guide the eye). The magenta dotted lines denote the position of the barrier maximum on the lower adiabatic surface, and the green dashed lines indicate the lowest four (adiabatically corrected) bound energy levels on the upper adiabatic surface.

As one nears the adiabatic barrier energy, there is a noticeable difference between the reflected quantum transition path times and those obtained from FSSH. Here, “barrier trajectories”, that is, classical trajectories whose energy is close to the barrier top, need long times to be reflected, while the nonlocal quantum mechanics smooths and shortens this classical maximum. When the initial wave packet is broad, these “barrier trajectories” contribute even when the incident mean wave packet energy is above the barrier so that the discrepancy appears over a longer range of energies. This classical time lag hardly appears in the transmitted times since the FSSH method gives transmission only when the incident trajectories are above the barrier.

Panels e and f of Figure 2 show the mean flight time differences at energies above the threshold for the opening of the excited state, allowing for the classically forbidden transmission to the upper surface (there was negligible reflected amplitude on the upper surface). Good agreement is observed between quantum and FSSH results everywhere except near the threshold, with the FSSH results again predicting shorter times than the numerically exact quantum results.

When the initial mean wave packet energy is between the top of the lower surface adiabatic barrier and the bottom of the well in the upper adiabatic surface, the transmission time using FSSH agrees well with the quantum results. In this energy regime, the reflection probability is small and quantum in origin and is therefore not observed using FSSH. Almost all trajectories avoid turning points.

Perhaps the most interesting energy regime is when the incident particle mean energy varies between the minimum of the upper adiabatic curve and the threshold of opening of the excited adiabatic surface (in the asymptotic region). One observes a series of peaks in the quantum mean flight time difference curves for both reflected and transmitted times corresponding to a significant slowing down of the motion of the particle at these energies. The peaks are broadened when the initial wave packet becomes wider in energy, and the scattering time becomes longer when the energy is closer to the threshold energy of the upper adiabatic surface. The four lowest bound energy levels of the upper adiabatic surface’s potential well (corrected with diagonal terms of the second-order nonadiabatic coupling) are also shown in Figure 2 and are consistent with the peaks in the mean time.

There are likely additional bound energy levels between the ones indicated in Figure 2 and the upper threshold. Hence one should not naively interpolate between the points shown in Figure 2 (and Figure 4) when the kinetic energy *E*_kin_ ranges between 0.01983 and about 0.02. In addition, FSSH results are not reported in some regions of panels a and b of Figures 2 and 4, where reflection on the lower surface is very unlikely. This is due to difficulties in converging FSSH calculations for reflection in these regions even when using around 10^6^ trajectories.

These time maxima indicate resonance trapping of the wave packet by the resonance states of the upper adiabatic well (the bound state energies are given in Supporting Information Section S1-C). The FSSH-generated mean times do not show these effects. They increase monotonically and lack the “bumps” corresponding to the resonances. This is due to the fact that FSSH does not account for the interference of waves as they slosh back and forth in the upper adiabatic well. When the incident wave packet is broadened (right panels) the resonance structure is smeared, yet the effect is noticeable. Also for the broad incident wave packets, the mean quantum time difference is much larger than predicted by FSSH.

Finally, when the incident energy is above the threshold energy of the excited adiabatic surface, one finds a significant drop in the transmitted and reflected times. This drop is reasonably well accounted for by the FSSH method.

It is also interesting to consider the flight time distributions in detail, so the transition path time distributions of reflected and transmitted particles at different incident mean energies and widths are plotted in Figure 3. The “QM” results in Figure 3 are the densities  from eq 3 in different channels plotted as functions of time, and the “FSSH” results are obtained by “binning” the distribution of flight times into small intervals.

**Figure 3:**
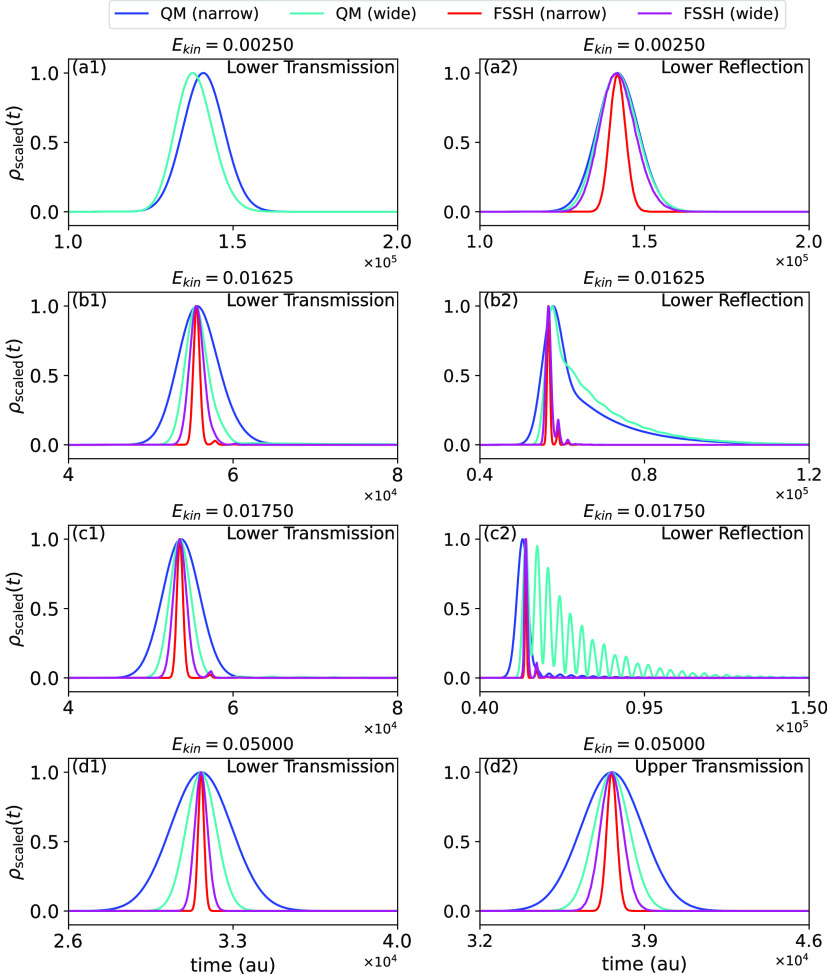
Flight time distributions computed by exact QM methods and the FSSH approximation. Panels a1 and a2 show time distributions (scaled such that the maximal value is unity) for transmission and reflection on the lower adiabatic surface respectively, for an initial mean kinetic energy which is below the ground electronic adiabatic barrier energy. FSSH results are shown only for classically allowed reflection. Panels b1 and b2 and c1 and c2 are in the resonance energy region, while panels d1 and d2 show transmission on the lower and upper surfaces, at an energy which is above the threshold of the upper adiabatic surface. In all panels, blue (cyan) lines: QM result for narrow width with α = 0.006 (or wide width with α = 0.030). Red (magenta) lines: FSSH results with α = 0.006 (α = 0.030).

As seen in panel a2 of Figure 3 for the deep tunneling regime and panels d1 and d2, even in the deep tunneling and high-energy regimes, where the FSSH method accurately reproduces mean flight times and probabilities, the numerically exact quantum flight time distributions are much broader than predicted by the FSSH method. This broadening accentuates the importance of broadening in time of quantum wave packets.

The resonance region is in the range of energies between the threshold of the upper adiabatic surface and the bottom of its well. Panels b1 and b2 of Figure 3 show the distributions when the incident mean energy is close to the lowest resonance energy, where the mean flight time, whether reflected or transmitted, shows a maximum. Panels c1 and c2 of Figure 3 show the transmitted and reflected distributions respectively at what may be considered an “anti-resonance” energy—that is, when the mean transmitted and reflected times show minima in panels a, c, and d of Figure 2. Consider first the transmitted time distribution. It is fairly broad in both cases, but shows no noticeable oscillations. At these energies, the classically allowed direct process dominates. Any resonance trapping is swamped by the direct process, yet at the resonance energy, one clearly sees that the width of the distribution on resonance is broader than off resonance. At these energies, reflection is a classically disallowed process so that the reflected time distribution is controlled by trapping in the well of the upper adiabatic potential. The QM broad-in-momentum curve (α = 0.03) shown in panel c2 of Figure 3 is especially interesting. The wave packet is sufficiently broad so as to have significant contributions from the two lowest resonance states, leading to a “beating” phenomenon between them (as also discussed and verified numerically in Supporting Information, Section S3). This beating is swamped in the transmitted distribution by the (classically allowed) direct transmission.

Interestingly, at some energies, such as at the “antiresonance” energy *E*_kin_ = 0.01750, the narrow fully quantum results are much closer to being simple Gaussians than the equivalent wider fully quantum ones. This is due to the fact that the wider-in-momentum wave packet overlaps with the two bound states and thus experiences resonance effects that are not present for the narrower one.

To complete the analysis it is also of interest to take a renewed look at the reflection and transmission probabilities, which should also show the resonance effect. Figure 4 shows these probabilities, which in the zero-width limit correspond to |*R*_1_|^2^, |*T*_1_|^2^, and |*T*_2_|^2^ as defined earlier. These are calculated by considering the fraction of trajectories that end in that channel for FSSH or the amount of wave function amplitude that ends in that channel for the exact quantum results.

**Figure 4:**
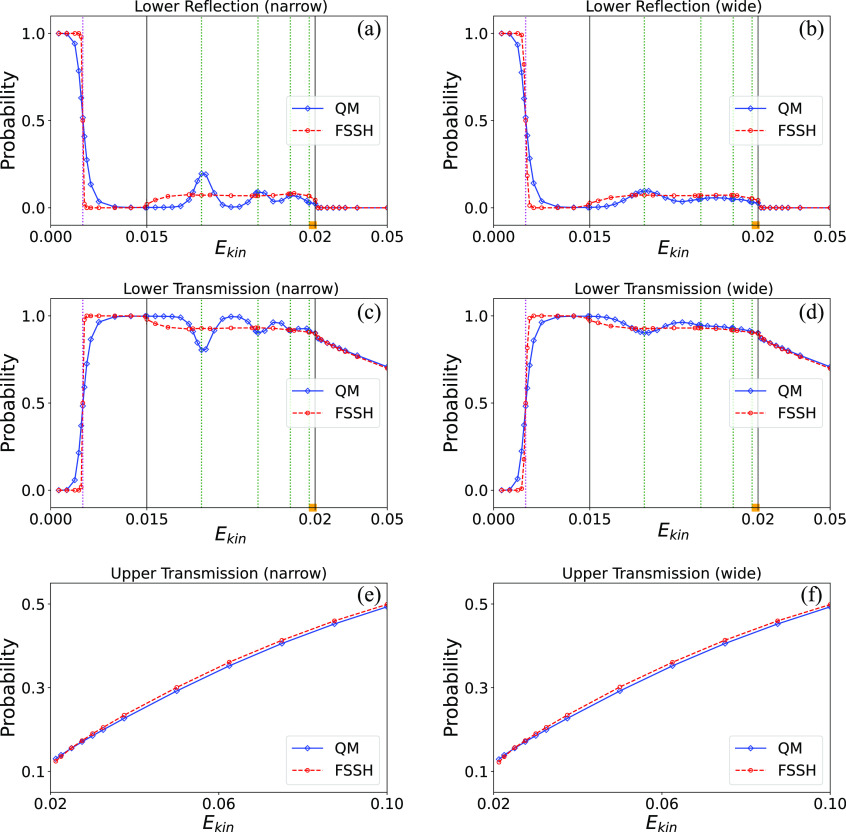
Energy-dependent transmission probabilities. The initial energy *E*_*kin*_ is the initial mean kinetic energy of the incident wave packet. Panels a and b show reflection probabilities on the lower surface corresponding to a narrow-in-momentum initial width (α = 0.006, left panel) and broad initial width (α = 0.03, right panel), respectively. Panels c and d show the same but for the transmission probability on the lower surface. Panels e and f likewise show transmission on the upper surface but with a different energy scale. In each panel, blue diamonds and red points represent QM results and FSSH results, respectively (blue solid and red dashed lines in each panel are used only to guide the eye). The barrier height energy of the lower adiabatic surface is denoted by the magenta dotted line. The location of the four lowest resonance energy levels on the adiabatically corrected excited adiabatic surface is indicated by the green dashed lines.

In Figure 4, one indeed observes oscillations in the transmission and reflection coefficients in the resonance region, which are somewhat smeared when using a broader-in-momentum initial distribution. Here too, the FSSH method notably fails to account for these. Reasonable agreement between the numerically exact quantum results and the FSSH approximation is found only when the momentum width of the initial wave packet is sufficiently large, so as to smear out the resonance oscillations. On the other hand, the FSSH method does succeed in obtaining a nonzero reflection coefficient in this energy region, where reflection is a classically disallowed process. In the high-energy region where classical effects dominate, the results are in good agreement with each other, and with those obtained by Tully.^37^

An important difference between the QM computation and the FSSH method is found for energies which are roughly equal to or lower than the height of the barrier of the ground adiabatic surface. Since FSSH misses any tunneling, it would predict thermal rate constants which are orders of magnitude too small at low enough temperatures.

This study presents a numerically exact computation of transition path flight time distributions for a model of an isolated electronic transition process, which sheds light on how coupling between electronic surfaces affects the flight times. Typically, when the coupling is important, it tends to increase the flight time, due to trapping, whether resonant or not, on the coupled electronic surfaces. A study of the flight times reveals resonance phenomena, which are observed through local maxima of the mean flight times and especially broadened flight time distributions.

The comparison between the QM and FSSH results is useful in elucidating where and how quantum effects are important in determining the mean times and the flight time distributions. We suspect that comparison with other quasi-classical approximate methods^31−33,56−62^ would reveal similar differences, as all such approximations do not include phases and quantum superposition. Although the present study was limited to what is arguably the simplest possible model, we expect that the effects considered here can sometimes become important when considering scattering with multiple surfaces or crossings, or in multidimensional systems where interferences cannot be ignored in the electronic transmission process.

The computations presented in this Letter were limited to one-dimensional systems. The extension of these results for one-dimensional avoided crossings to higher-dimensional equivalents such as conical intersections is not trivial. Already for the one-dimensional computation, the determination of FSSH flight time distributions necessitated  trajectories. Quantum interference effects, which are especially important when considering conical intersections, should affect the flight time distributions as they do in the present one-dimensional system. It has also been shown that geometric phase effects lead to *quenching* of tunneling in model system studies at low energies.^63^ FSSH for example, has been shown to somewhat incorporate geometric phase effects.^64^ It is therefore especially interesting to expand the present flight time computation to the study of systems with conical intersections.

FSSH has been expanded upon many times over the years, such as with corrections for decoherence effects^38,39,41,42,65^ and tunneling effects,^66^ as well as “phase-corrected” FSSH methods.^40^ However, these corrections, as shown in some detail in the Supporting Information, are insufficient. Even the phase-corrected methods do not account for the phase effects of *nuclear motion* and so cannot produce the resonances and their impact on the flight time distributions. These observations indicate that semiclassical methods which do incorporate nuclear motion phase information may be very helpful. At the same time these are much more expensive to implement, so the method to be used would probably depend on the system chosen to be studied.

The resonance effects and other time-domain phenomena presented show FSSH results will match fully quantum ones more closely if one broadens the incident wavepacket considerably such that quantum coherence effects are diminished. We expect that a similar conclusion applies to other trajectory-based approximate methods.^31−33,56−62,65,66^ It will be interesting to see whether it is possible to further improve upon surface hopping,^37−42,65,66^ phase space mapping dynamics approaches,^31−33,59−62^ and other trajectory-based nonadiabatic methods so that they can capture the type of resonance effects described in this Letter.

## Supporting Information Available

Section S1, methodology and further details, including quantum calculations via the split-operator method, the discrete variable representation method, bound state energies, and numerical parameters used in quantum mechanics calculations; Section S2, methodology and further details of surface hopping, with fewest switches surface hopping and its variants, comparison of different types of surface hopping approximations, and numerical parameters used in surface hopping calculations; Section S3, the beating phenomenon in the resonance region; and Section S4, the impact of wave packet widths on flight times (PDF)

## References

[ref1] Hauge E.; Støvneng J. Tunneling Times: a Critical Review. Rev. Mod. Phys. 1989, 61, 917–936. 10.1103/RevModPhys.61.917.

[ref2] Hauge E. H. In Tunneling and its Implications: Proceedings Of The Adriatico Research Conference; Mugnai D., Ranfagni A., Schulman L. S., Eds.; World Scientific Singapore, 1997; pp 1–17; 10.1142/9789814530354.

[ref3] Lozovik Y. E.; Filinov A. Transmission Times of Wave Packets Tunneling Through Barriers. J. Exp. Theor. Phys. 1999, 88, 1026–1035. 10.1134/1.558886.

[ref4] Muga J. G.; Leavens C. R. Arrival Time in Quantum Mechanics. Phys. Rep. 2000, 338, 353–438. 10.1016/S0370-1573(00)00047-8.

[ref5] McDonald C.; Orlando G.; Vampa G.; Brabec T. Tunneling Time, What is Its Meaning?. J. Phys. Conf. Ser. 2015, 594, 012019 10.1088/1742-6596/594/1/012019.

[ref6] Dumont R. S.; Rivlin T.; Pollak E. The Relativistic Tunneling Flight Time May Be Superluminal, But It Does Not Imply Superluminal Signaling. New J. Phys. 2020, 22, 093060 10.1088/1367-2630/abb515.

[ref7] Fuhrmanek A.; Lance A. M.; Tuchendler C.; Grangier P.; Sortais Y. R.; Browaeys A. Imaging a Single Atom in a Time-of-Flight Experiment. New J. Phys. 2010, 12, 053028 10.1088/1367-2630/12/5/053028.

[ref8] Du J.-J.; Li W.-F.; Wen R.-J.; Li G.; Zhang T.-C. Experimental Investigation of the Statistical Distribution of Single Atoms in Cavity Quantum Electrodynamics. Laser Phys. Lett. 2015, 12, 065501 10.1088/1612-2011/12/6/065501.

[ref9] Schulman L. S. In Time in Quantum Mechanics; Muga J. G., Mayato R. S., Egusquiza Í. L., Eds.; Springer Berlin Heidelberg: 2008; pp 99–120; 10.1007/3-540-45846-8_4.

[ref10] Muga J. G. In Time in Quantum Mechanics; Muga J. G., Mayato R. S., Egusquiza Í. L., Eds.; Springer Berlin Heidelberg: 2008; pp 29–68; 10.1007/3-540-45846-8_2.

[ref11] Miret-Artés S.; Dumont R. S.; Rivlin T.; Pollak E. The Influence of the Symmetry of Identical Particles on Flight Times. Entropy 2021, 23, 1675 10.3390/e23121675. 34945981 PMC8700582

[ref12] Dumont R. S.; Marchioro T. II Tunneling-Time Probability Distribution. Phys. Rev. A 1993, 47, 85–97. 10.1103/PhysRevA.47.85. 9908897

[ref13] Baute A. D.; Egusquiza Í. L.; Muga J. G. Time-of-Arrival Distributions for Interaction Potentials. Phys. Rev. A 2001, 64, 012501 10.1103/PhysRevA.64.012501.

[ref14] Egusquiza I. L.; Muga J. G.; Baute A. D. In Time in Quantum Mechanics; Muga J. G., Mayato R. S., Egusquiza Í. L., Eds.; Springer Berlin Heidelberg, 2008; pp 305–332; 10.1007/978-3-540-73473-4_10.

[ref15] Ruschhaupt A.; Muga J. G.; Hegerfeldt G. C. In Time in Quantum Mechanics; Muga J. G., Ruschhaupt A., Campo A., Eds.; Springer Berlin Heidelberg: 2009; Vol. 2, pp 65–96; 10.1007/978-3-642-03174-8_4.

[ref16] Rivlin T.; Pollak E.; Dumont R. S. Determination of the Tunneling Flight Time as the Reflected Phase Time. Phys. Rev. A 2021, 103, 012225 10.1103/PhysRevA.103.012225.

[ref17] Rivlin T.; Pollak E.; Dumont R. S. Comparison of a Direct Measure of Barrier Crossing Times with Indirect Measures Such as the Larmor Time. New J. Phys. 2021, 23, 063044 10.1088/1367-2630/ac047b.

[ref18] Shafir D.; Soifer H.; Bruner B. D.; Dagan M.; Mairesse Y.; Patchkovskii S.; Ivanov M. Y.; Smirnova O.; Dudovich N. Resolving the Time When an Electron Exits a Tunnelling Barrier. Nature 2012, 485, 343–346. 10.1038/nature11025. 22596157

[ref19] Landsman A. S.; Keller U. Attosecond Science and the Tunnelling Time Problem. Phys. Rep. 2015, 547, 1–24. 10.1016/j.physrep.2014.09.002.

[ref20] Torlina L.; Morales F.; Kaushal J.; Ivanov I.; Kheifets A.; Zielinski A.; Scrinzi A.; Muller H. G.; Sukiasyan S.; Ivanov M.; et al. Interpreting Attoclock Measurements of Tunnelling Times. Nat. Phys. 2015, 11, 503–508. 10.1038/nphys3340.

[ref21] Ramos R.; Spierings D.; Racicot I.; Steinberg A. Measurement of the Time Spent by a Tunnelling Atom Within the Barrier Region. Nature 2020, 583, 529–532. 10.1038/s41586-020-2490-7. 32699398

[ref22] Spierings D. C.; Steinberg A. M. In Optical, Opto-Atomic, and Entanglement-Enhanced Precision Metrology II; Shahriar S. M., Scheuer J., Eds.; SPIE Bellingham: 2020; Vol. 11296; p 112960F; 10.1117/12.2552583.

[ref23] Kheifets A. S. The Attoclock and the Tunneling Time Debate. J. Phys. B-At. Mol. Opt. 2020, 53, 072001 10.1088/1361-6455/ab6b3b.

[ref24] Satya Sainadh U.; Sang R. T.; Litvinyuk I. V. Attoclock and the Quest for Tunnelling Time in Strong-Field Physics. J. Phys. Photonics 2020, 2, 042002 10.1088/2515-7647/aba009.

[ref25] Spierings D. C.; Steinberg A. M. Observation of the Decrease of Larmor Tunneling Times with Lower Incident Energy. Phys. Rev. Lett. 2021, 127, 133001 10.1103/PhysRevLett.127.133001. 34623833

[ref26] Petersen J.; Pollak E. Tunneling Flight Time, Chemistry, and Special Relativity. J. Phys. Chem. Lett. 2017, 8, 4017–4022. 10.1021/acs.jpclett.7b02018. 28792772

[ref27] Pollak E.; Miret-Artés S. Time Averaging of Weak Values—Consequences for Time-Energy and Coordinate-Momentum Uncertainty. New J. Phys. 2018, 20, 073016 10.1088/1367-2630/aad020.

[ref28] Petersen J.; Pollak E. Quantum Coherence in the Reflection of Above Barrier Wavepackets. J. Chem. Phys. 2018, 148, 074111 10.1063/1.5019221. 29471663

[ref29] Ianconescu R.; Pollak E. Determination of the Mean Tunneling Flight Time in the Büttiker-Landauer Oscillating-Barrier Model as the Reflected Phase Time. Phys. Rev. A 2021, 103, 042215 10.1103/PhysRevA.103.042215.

[ref30] Heller E. R.; Richardson J. O. Instanton Formulation of Fermi’s Golden Rule in the Marcus Inverted Regime. J. Chem. Phys. 2020, 152, 034106 10.1063/1.5137823. 31968950

[ref31] He X.; Wu B.; Gong Z.; Liu J. Commutator Matrix in Phase Space Mapping Models for Nonadiabatic Quantum Dynamics. J. Phys. Chem. A. 2021, 125, 6845–6863. 10.1021/acs.jpca.1c04429. 34339600

[ref32] Liu J.; He X.; Wu B. Unified Formulation of Phase Space Mapping Approaches for Nonadiabatic Quantum Dynamics. Acc. Chem. Res. 2021, 54, 4215–4228. 10.1021/acs.accounts.1c00511. 34756027

[ref33] He X.; Wu B.; Shang Y.; Li B.; Cheng X.; Liu J. New Phase Space Formulations and Quantum Dynamics Approaches. Wiley Interdiscip. Rev. Comput. Mol. Sci. 2022, e1619 10.1002/wcms.1619.

[ref34] Ansari I. M.; Heller E. R.; Trenins G.; Richardson J. O. Instanton Theory for Fermi’s Golden Rule and Beyond. Philos. Trans. R. Soc. A 2022, 380, 20200378 10.1098/rsta.2020.0378. PMC8958279 35341312

[ref35] Dion C. M.; Hashemloo A.; Rahali G. Program for Quantum Wave-Packet Dynamics with Time-Dependent Potentials. Comput. Phys. Commun. 2014, 185, 407–414. 10.1016/j.cpc.2013.09.012.

[ref36] Colbert D. T.; Miller W. H. A Novel Discrete Variable Representation for Quantum Mechanical Reactive Scattering Via the S-Matrix Kohn Method. J. Chem. Phys. 1992, 96, 1982–1991. 10.1063/1.462100.

[ref37] Tully J. C. Molecular Dynamics with Electronic Transitions. J. Chem. Phys. 1990, 93, 1061–1071. 10.1063/1.459170.

[ref38] Zhu C.; Jasper A. W.; Truhlar D. G. Non-Born–Oppenheimer Trajectories with Self-Consistent Decay of Mixing. J. Chem. Phys. 2004, 120, 5543–5557. 10.1063/1.1648306. 15267430

[ref39] Subotnik J. E.; Shenvi N. A New Approach to Decoherence and Momentum Rescaling in the Surface Hopping Algorithm. J. Chem. Phys. 2011, 134, 024105 10.1063/1.3506779. 21241078

[ref40] Shenvi N.; Subotnik J. E.; Yang W. Phase-Corrected Surface Hopping: Correcting the Phase Evolution of the Electronic Wavefunction. J. Chem. Phys. 2011, 135, 024101 10.1063/1.3603447. 21766919

[ref41] Jaeger H. M.; Fischer S.; Prezhdo O. V. Decoherence-Induced Surface Hopping. J. Chem. Phys. 2012, 137, 22A545 10.1063/1.4757100. 23249082

[ref42] Wang L.; Akimov A.; Prezhdo O. V. Recent Progress in Surface Hopping: 2011–2015. J. Phys. Chem. Lett. 2016, 7, 2100–2112. 10.1021/acs.jpclett.6b00710. 27171314

[ref43] Prezhdo O. V.; Rossky P. J. Evaluation of Quantum Transition Rates from Quantum-Classical Molecular Dynamics Simulations. J. Chem. Phys. 1997, 107, 5863–5878. 10.1063/1.474312.

[ref44] Craig C. F.; Duncan W. R.; Prezhdo O. V. Trajectory Surface Hopping in the Time-Dependent Kohn-Sham Approach for Electron-Nuclear Dynamics. Phys. Rev. Lett. 2005, 95, 163001 10.1103/PhysRevLett.95.163001. 16241791

[ref45] Fabiano E.; Keal T.; Thiel W. Implementation of Surface Hopping Molecular Dynamics Using Semiempirical Methods. Chem. Phys. 2008, 349, 334–347. 10.1016/j.chemphys.2008.01.044.

[ref46] Barbatti M. Nonadiabatic Dynamics with Trajectory Surface Hopping Method. Wiley Interdiscip. Rev. Comput. Mol. Sci. 2011, 1, 620–633. 10.1002/wcms.64.

[ref47] Jain A.; Herman M. F.; Ouyang W.; Subotnik J. E. Surface Hopping, Transition State Theory and Decoherence. I. Scattering Theory and Time-Reversibility. J. Chem. Phys. 2015, 143, 134106 10.1063/1.4930548. 26450291

[ref48] Subotnik J. E.; Jain A.; Landry B.; Petit A.; Ouyang W.; Bellonzi N. Understanding the Surface Hopping View of Electronic Transitions and Decoherence. Annu. Rev. Phys. Chem. 2016, 67, 387–417. 10.1146/annurev-physchem-040215-112245. 27215818

[ref49] Long R.; Prezhdo O. V.; Fang W. Nonadiabatic Charge Dynamics in Novel Solar Cell Materials. Wiley Interdiscip. Rev. Comput. Mol. Sci. 2017, 7, e1305 10.1002/wcms.1305.

[ref50] Agostini F.; Curchod B. F. E. Chemistry Without the Born–Oppenheimer Approximation. Philos. Trans. R. Soc. A 2022, 380, 20200375 10.1098/rsta.2020.0375. PMC8958276 35341309

[ref51] Hammes-Schiffer S. Theoretical Perspectives on Non-Born–Oppenheimer Effects in Chemistry. Philos. Trans. R. Soc. A 2022, 380, 20200377 10.1098/rsta.2020.0377. 35341306

[ref52] Avagliano D.; Lorini E.; González L. Sampling Effects in Quantum Mechanical/Molecular Mechanics Trajectory Surface Hopping Non-adiabatic Dynamics. Philos. Trans. R. Soc. A 2022, 380, 20200381 10.1098/rsta.2020.0381. PMC8958275 35341304

[ref53] Coonjobeeharry J.; Spinlove K. E.; Sanz Sanz C.; Sapunar M.; Došlić N.; Worth G. A. Mixed-Quantum-Classical or Fully-Quantized Dynamics? A Unified Code to Compare Methods. Philos. Trans. R. Soc. A 2022, 380, 20200386 10.1098/rsta.2020.0386. 35341308

[ref54] Baer M. Beyond Born-Oppenheimer: Electronic Nonadiabatic Coupling Terms and Conical Intersections; John Wiley & Sons Hoboken: 2006; 10.1002/0471780081.

[ref55] Ibele L. M.; Curchod B. F. E. A Molecular Perspective on Tully Models for Nonadiabatic Dynamics. Phys. Chem. Chem. Phys. 2020, 22, 15183–15196. 10.1039/D0CP01353F. 32582887

[ref56] Kapral R.; Ciccotti G. Mixed Quantum-Classical Dynamics. J. Chem. Phys. 1999, 110, 8919–8929. 10.1063/1.478811.

[ref57] Wan C.-C.; Schofield J. Mixed Quantum-Classical Molecular Dynamics: Aspects of the Multithreads Algorithm. J. Chem. Phys. 2000, 113, 7047–7054. 10.1063/1.1313525.

[ref58] Ando K.; Santer M. Mixed Quantum-Classical Liouville Molecular Dynamics without Momentum Jump. J. Chem. Phys. 2003, 118, 10399–10406. 10.1063/1.1574015.

[ref59] Miller W. H.; Cotton S. J. Classical Molecular Dynamics Simulation of Electronically Non-Adiabatic Processes. Faraday Discuss. 2016, 195, 9–30. 10.1039/C6FD00181E. 27828549

[ref60] Cotton S. J.; Miller W. H. Trajectory-Adjusted Electronic Zero Point Energy in Classical Meyer-Miller Vibronic Dynamics: Symmetrical Quasiclassical Application to Photodissociation. J. Chem. Phys. 2019, 150, 194110 10.1063/1.5094458. 31117780

[ref61] He X.; Gong Z.; Wu B.; Liu J. Negative Zero-Point-Energy Parameter in the Meyer-Miller Mapping Model for Nonadiabatic Dynamics. J. Phys. Chem. Lett. 2021, 12, 2496–2501. 10.1021/acs.jpclett.1c00232. 33667108

[ref62] Saller M. A. C.; Lai Y.; Geva E. An Accurate Linearized Semiclassical Approach for Calculating Cavity-Modified Charge Transfer Rate Constants. J. Phys. Chem. Lett. 2022, 13, 2330–2337. 10.1021/acs.jpclett.2c00122. 35245071

[ref63] Ryabinkin I. G.; Joubert-Doriol L.; Izmaylov A. F. Geometric Phase Effects in Nonadiabatic Dynamics Near Conical Intersections. Acc. Chem. Res. 2017, 50, 1785–1793. 10.1021/acs.accounts.7b00220. 28665584

[ref64] Gherib R.; Ryabinkin I. G.; Izmaylov A. F. Why Do Mixed Quantum-Classical Methods Describe Short-Time Dynamics Through Conical Intersections So Well? Analysis of Geometric Phase Effects. J. Chem. Theory Comput. 2015, 11, 1375–1382. 10.1021/acs.jctc.5b00072. 26574349

[ref65] Cheng S. C.; Zhu C.; Liang K. K.; Lin S. H.; Truhlar D. G. Algorithmic Decoherence Time for Decay-of-Mixing Non-Born-Oppenheimer Dynamics. J. Chem. Phys. 2008, 129, 024112 10.1063/1.2948395. 18624521

[ref66] Zhu C.; Nobusada K.; Nakamura H. New Implementation of the Trajectory Surface Hopping Method with Use of the Zhu-Nakamura Theory. J. Chem. Phys. 2001, 115, 3031–3044. 10.1063/1.1386811.

