# Supplementary material for: Transition Path Flight Times and Nonadiabatic Electronic Transitions
Source: J Phys Chem Lett. 2022 Jul 25;13(30):6966–74. doi: 10.1021/acs.jpclett.2c01425 (PMC9358656; doi:10.1021/acs.jpclett.2c01425)
Supplement: Supplementary file 1 — jz2c01425_si_001.pdf [file jz2c01425_si_001.pdf]

Supporting Information: Transition Path Flight Times and Nonadiabatic Electronic Transitions

Xin He,[†,¶] Baihua Wu,[†,¶] Tom Rivlin,[‡] Jian Liu,[*,†] and Eli Pollak[*,‡]

†*Beijing National Laboratory for Molecular Sciences, Institute of Theoretical and Computational Chemistry, College of Chemistry and Molecular Engineering, Peking University, Beijing 100871, China*

‡*Chemical and Biological Physics Department, Weizmann Institute of Science, 76100 Rehovot, Israel*

¶*These authors contributed equally to this paper.*

E-mail: jianliupku@pku.edu.cn; eli.pollak@weizmann.ac.il

The Supporting Information contains four Sections. In Section S1, we discuss the numerical methods used to produce the numerically-exact quantum mechanical results: the split-operator method and the discrete variable representation method (with absorption potentials). We also present the values of the calculated bound state energies (with and without nonadiabatic correction), and the numerical parameters used in the quantum calculations. In Section S2, we present more details on the surface hopping simulations: we explain the fewest switches surface hopping algorithm and some of its variants, including decoherence-induced surface hopping[1] (DISH) and phase-corrected fewest switches surface hopping[2] (PC-FSSH). We also compare flight times and scattering probabilities generated using FSSH variants to regular FSSH and quantum results, and provide numerical parameters used in the main calculations. In Section S3, we provide further information on the 'beating' phenomenon observed in the resonance region. Finally in Section S4, we discuss how the mean flight time varies as a function of the width parameter of the incident Gaussian wavepacket at selected energies in the quantum and FSSH regimes.

S1: Methodology and further details: Quantum calculations

S1-A: The Split-Operator method

The Split-Operator (SO) method is a fully-quantum, exact numerical method. It can be performed either diabatically or adiabatically. Here only the diabatic representation was used. The method was implemented using the code *wavepacket*,[3] which was modified to be able to propagate a wave packet over a two-level system with diabatic off-diagonal couplings, and to print the wavefunction as a function of time at certain x-coordinates.

The principle behind the method is that at each time step, two fast Fourier transforms (FFTs) are used to translate between the representations of the wavefunction in x-space and k-space – one FFT and one inverse-FFT is performed per time step. In k-space, the kinetic term of the full diabatic Hamiltonian is propagated forward in time, and in x-space, the potential term of the Hamiltonian is propagated. As such, no derivatives need be calcu-

lated. Applying each operator involves only multiplication. (The full exponentiated matrix elements of the two-level potential energy matrix are calculated explicitly and input directly to facilitate this.)

Using this method, one obtains the full two-component wavefunction $\mathbf{\Psi}(x,t)$ at every discretized value of the x coordinate in the range, and at each time step. This is then used to calculate the integrals discussed in the main text to obtain transmission and reflection times and probabilities on the upper and lower surfaces. One notable drawback of the method is that it creates periodic boundary conditions at the edges of the x-space grid, meaning a very large grid must be used to ensure the wavefunction amplitudes are correct in the asymptotic regions where the screens are placed, as absorbing potentials were not used.

S1-B: The Discrete Variable Representation method

The discrete variable representation (DVR) has been proposed as an efficient approach to solve low-dimensional quantum problems in a numerically exact manner. Here we adopt the sinc-DVR developed by Colbert and Miller.[4] In this DVR basis, $|\chi_\alpha\rangle$ is localized in the neighborhood of the discrete coordinate points x_α, the matrix representations for its nuclear operator ∇ and ∇^2 (the Laplacian operator) can be written:

$$D_{\alpha\beta} = \langle\chi_\alpha|\nabla|\chi_\beta\rangle \doteq \sum_{j,k=1}^{N} \frac{2}{N+1} \sin\frac{j\alpha\pi}{N+1} \sin\frac{k\beta\pi}{N+1} \,\mathrm{Mod}(j-k,2)\frac{4}{L}\frac{jk}{j^2-k^2}, \tag{1}$$

$$D^{(2)}_{\alpha\beta} = \langle\chi_\alpha|\nabla^2|\chi_\beta\rangle \doteq -(\frac{\pi}{\Delta x})^2 \begin{cases} -\frac{1}{3} + \frac{1}{6(N+1)^2} - \frac{1}{2(N+1)^2\sin^2(\alpha\pi/(N+1))} & \alpha = \beta \\ \frac{2(-1)^{\alpha-\beta}}{(N+1)^2}\frac{\sin(\alpha\pi/(N+1))\sin(\beta\pi/(N+1))}{(\cos(\alpha\pi/(N+1))-\cos(\beta\pi/(N+1)))^2} & \alpha \neq \beta \end{cases}, \tag{2}$$

and the potential surface is diagonal at the discrete points $\{x_\alpha\}$, since $V_{\alpha\beta} = V(x_\alpha)\delta_{\alpha\beta}$. These formulations can be naturally extended to the one-dimensional, multi-electronic quantum problem. Since the electronic basis is specified with a given representation, i.e., the basis is chosen as $|\phi_i\rangle, i = 1,2$ for a two-level system, in a composite basis $|\chi_\alpha\rangle \otimes |\phi_i\rangle$, the full

Hamiltonian should be built as

$$\begin{aligned} H_{jk}^{\alpha\beta} = -\frac{\hbar^2}{2M}\Big(& D_{\alpha\beta}^{(2)}\delta_{jk} + (d_{jk}(x_\alpha) + d_{jk}(x_\beta)) \cdot D_{\alpha\beta} \\ & + \sum_l d_{jl}(x_\alpha) d_{lk}(x_\alpha)\delta_{\alpha\beta}\Big) + V_{jk}(x_\alpha)\delta_{\alpha\beta}, \end{aligned} \tag{3}$$

where i, j label electronic surfaces, α, β label nuclear discrete points, V_{jk} is an element of the potential matrix between the i-th and j-th electronic surfaces, and $d_{jk} = \langle\phi_j|\partial_R\phi_k\rangle$ is the first-order nonadiabatic vector between the i-th and j-th electronic surfaces. Both of these are diagonal with nuclear DOFs, where,

$$\begin{aligned} V_{jk}^{\alpha\beta} &= V_{jk}(x_\alpha)\delta_{\alpha\beta} \\ d_{jk}^{\alpha\gamma} &= d_{jk}(x_\alpha)\delta_{\alpha\gamma} \end{aligned}. \tag{4}$$

In simulations of wave packet propagation, this provides a stable algorithm for calculating the eigenvalues and eigenfunctions of $H_{jk}^{\alpha\beta}$ in a DVR basis. The eigenvalue equation may be denoted as $\mathbf{HT} = \mathbf{ET}$, where $\mathbf{E}$ is a diagonal matrix of the eigenvalues of $\mathbf{H}$, so that the exact propagator is obtained as

$$\mathbf{U} = e^{-i\mathbf{H}t} = \mathbf{T}e^{-i\mathbf{E}t}\mathbf{T}^\dagger. \tag{5}$$

This may be numerically costly if the number of grid points needed for the nuclear DOFs increases. However, it is possible to perform an exact propagation of the wave packet by projecting onto the DVR eigenvectors.

Due to resonance effects, a tailing phenomenon occurs when the wave packet leaves the upper potential well, which means that the scattering time distribution cannot decay completely unless the simulation is long enough. However, the actual boundaries in numerically exact quantum mechanically simulations cannot be infinite, while the reflection boundary condition (e.g., for sinc-DVR) or periodic boundary conditions (e.g., exp-DVR) are employed.

In order to efficiently propagate the wave packet for long enough times, one can add an absorption potential $-iV_{abs}(x)$ in the boundary region, where $V_{abs}(x)$ is a positive-definite real function. In this work, we employ an absorption potential with the form

$$V_{abs}(x) = C_1(e^{-C_2(x-x_{\min})^2} + e^{-C_2(x-x_{\max})^2}), \tag{6}$$

where $C_1 = 1$ and $C_2 = 0.01$. In the DVR calculations, adding the absorption potential removes the Hermiticity of the full Hamiltonian, so the eigenproblem should be solved in both the left and right eigenvector spaces, i.e., $\mathbf{H}\mathbf{T}_r = \mathbf{T}_r\mathbf{E}$, $\mathbf{T}_l^\dagger\mathbf{H} = \mathbf{E}\mathbf{T}_l^\dagger$ (with $\mathbf{T}_l$ and $\mathbf{T}_r$ respectively containing the left and right eigenvectors of the now non-Hermitian matrix $\mathbf{H}$). Defining $\mathbf{S} = \mathbf{T}_l^\dagger\mathbf{T}_r$ as the overlap matrix between the left and right eigenvector spaces of $\mathbf{H}$, the exact propagator can be constructed, and

$$\mathbf{U} = e^{-i\mathbf{H}t} = \mathbf{T}_r\mathbf{S}^{-1}\exp[-i\mathbf{E}t]\mathbf{T}_l^\dagger. \tag{7}$$

is used instead of eq 5.

S1-C: Bound state energies

Bound state energies can be calculated adiabatically by solving the time-independent Schrödinger equation for the PES in question. This was done adiabatically using the DVR-based program Duo,[5] and with our own DVR-based code for the one-dimensional system, with the two methods producing the same outputs. To calculate the non-adiabatic corrections to these energies, more careful considerations needed to be made.

The Born-Oppenheimer correction for the n-th state is

$$\Delta E_n(R) = -\frac{\hbar^2}{2M}F_{nn}(R), \tag{8}$$

where $F_{nn}(R) = \langle\phi_n|\frac{\partial^2}{\partial R^2}|\phi_n\rangle$ is the n-th diagonal term of the second-order nonadiabatic

coupling. One can show that

$$F_{11}(R) = F_{22}(R) = -d_{12}^2(R) \tag{9}$$

for the two-electronic-surface system. Therefore, the corrected PES is

$$E_n^{(\mathrm{corr})}(R) = E_n(R) + \frac{d_{12}^2(R)}{2M}, \quad n = 1, 2, \tag{10}$$

and the bound energies with Born-Oppenheimer corrections can be obtained using the DVR method, where the matrix elements are

$$\begin{aligned} H_{nn}^{\alpha\beta} &= -\frac{\hbar^2}{2M} D_{\alpha\beta}^{(2)} + E_n^{(\mathrm{corr})}(x_\alpha)\delta_{\alpha\beta} \\ &= -\frac{\hbar^2}{2M}(D_{\alpha\beta}^{(2)} + d_{21}(x_\alpha)d_{12}(x_\alpha)\delta_{\alpha\beta}) + E_n(x_\alpha)\delta_{\alpha\beta}, \quad n = 1, 2. \end{aligned} \tag{11}$$

Eq. 11 is actually the corresponding element of the electronic diagonal block of Eq. 3, where the nonadiabatic effect is already considered. Otherwise, when ignoring d_{12} in Eq. 11, it provides the bound energies of the original PES in the case where the Born-Oppenheimer approximation is valid.

- *Bound state energies calculated with* DUO[5] *adiabatically (in atomic units):* (0.016367, 0.018235, 0.019212, 0.019808)
- *Bound state energies calculated by our DVR-based code with nonadiabatic corrections:* (0.016621, 0.018300, 0.019262, 0.019823)

S1-D: Numerical parameters in quantum mechanics calculations (in atomic units)

The center of the initial wave packet was $x_0 = -77.8617$ and the screens were placed at $Y_i = \pm 145.723$. For the split-operator method, $\Delta t = 2$, $\Delta x \approx 0.003$, $x_{\min} \approx 500$, $x_{\max} \approx 500$, $t_{\max} \approx 10^5$. For the DVR method, the boundary was chosen with $x_{\min} = -2|Y_i|$ and $x_{\max} = 2|Y_i|$. $N_x = 6000$ grids were used per electronic surface for DVR discretization. An absorption potential with $C_1 = 1, C_2 = 0.01$ was used, centered in the boundary side.

S2: Methodology and further details: Surface hopping

S2-A: Fewest Switches Surface Hopping and its variants

The numerically exact quantum flight times were compared with surface hopping mixed quantum-classical approximations. We employed the well-known Tully fewest-switches surface hopping algorithm,[6] which can be described as follows:

1. The initial active state of a trajectory $i_{act}(0)$ is chosen, and the initial electronic amplitude is given by $c_n(0) = \delta_{n,i_{act}(0)}$, while the nuclear positions and momenta are sampled from the (Gaussian) Wigner distribution function of the initial (Gaussian) wave packet.

2. At each Δt time step, the electronic amplitude c_n is propagated using the Schrödinger equation $i\hbar\dot{c}_n(t) = \sum_m H_{nm}c_m(t)$. Here H_{nm} is the adiabatic Hamiltonian: $H_{nm} = E_n(R)\delta_{nm} - i\hbar d_{nm}(R) \cdot M^{-1}P$, R and P are respectively the nuclear position and momentum, and $E_n(R)$ and $d_{nm}(R)$ are respectively the adiabatic potential energy surfaces and nonadiabatic coupling between nth and mth state.

3. At each Δt time step, the fewest switches algorithm is applied to update the current active state $i_{act}(t + \Delta t)$: the 'hopping' probabilities from the current state $i_{act}(t)$ to other states are calculated using

$$w_{i_{act}\to j} = -\frac{2\mathrm{Im}[c_j c^*_{i_{act}} H_{i_{act}j}]}{c_{i_{act}} c^*_{i_{act}}}\Delta t \tag{12}$$

   In the simulation, the hopping probability $w_{i_{act}\to i_{act}}$ is set to zero, and all probabilities are truncated into the $[0, 1]$ range.[6,7] Hopping events can be made using the Monte Carlo algorithm by generating a uniform random number ξ between $[0, 1]$ and determining which subsection the random number lies in. For example, if ξ falls in $[\sum_{j=1}^{k-1} w_j, \sum_{j=1}^{k} w_j]$, then a hop to the k-th state will occur. When a hopping is attempted, the nuclear momentum is corrected along the direction of the nonadiabatic vector to ensure conservation of the total energy. If energy conservation cannot be satisfied, such hopping events are deprecated (this is known as frustrated hopping). Once hopping succeeds, $i_{act}(t + \Delta t)$ is updated to the current state.

4. At each Δt time step, the nuclear motion on i_{act}th adiabatic surface is evolved according to classical dynamics:

$$\begin{aligned} \dot{R}(t) &= M^{-1}P(t) \\ \dot{P}(t) &= -\partial_R E_{i_{act}}(R(t)) \end{aligned} \tag{13}$$

5. Steps 2-4 are repeated until the trajectory leaves the interaction region.

6. The algorithm is repeated for enough trajectories to sufficiently construct the statistics of the desired observable.

Among other problems, Tully's original algorithm cannot account for decoherence. As such, many authors since Tully have proposed adjustments to his method to account for these effects and others.[1,2,8–10] Here we briefly introduce two such surface-hopping variants (more details can be found elsewhere[1,2]), denoting Tully's original method as just FSSH.

The first variant is decoherence-induced surface hopping (DISH),[1] which includes both quantum mechanical branching in the nuclear degrees of freedom and loss of coherence in the electronic degrees of freedom. This loss of coherence is achieved by stochastic modeling of the (time-dependent) Schrödinger and master equations in an open system. The key difference from FSSH is that hopping only occurs when an estimated 'decoherence time' (generated from a Poisson distribution) is less than the coherence time since the last decoherence event. Furthermore, in DISH, a quantum formalism (i.e., $|c_n|^2$) rather than a classical formalism (i.e., the proportion of classical trajectories) is used for counting populations in channels.

The second variant is phase-corrected fewest switches surface hopping[2] (PC-FSSH), which tracks a phase difference between two Gaussian wave packets in addition to position and momentum information. This modification has been proven to be valid in the description of oscillatory behavior in the scattering problem, and it is claimed that the method can account for partial decoherence. The difference between PC-FSSH and FSSH is minor: PC-FSSH uses a modified Hamiltonian to propagate the electronic amplitude.

S2-B: Comparison of different types of surface hopping

In the main text, we compare flight times calculated using fully-quantum methods and using a (semi-)classical surface hopping method. It has been shown that FSSH is incapable of capturing resonance behavior in the 'well' region (i.e., for E_{kin} in the range $[0.015, 0.020]$). In this subsection, we investigate whether DISH and PC-FSSH can improve on these results.

Supporting Fig. 1 shows the reflection and transmission probabilities on the lower surface as functions of kinetic energy E_{kin} in the resonance region. It shows that DISH does worse than even FSSH when estimating transmission and reflection probabilities, let alone capturing resonance behavior. Whilst PC-FSSH qualitatively displays oscillations, these are

out of phase with the true resonance peaks and nowhere near as large in amplitude.

Supporting Fig. 2 details reflected and transmitted flight times on the lower surface as functions of kinetic energy E_{kin}. Here, DISH is a clear improvement on FSSH for reflected flight times on the lower surface when $E_{kin} > 0.018$, but the same cannot be said for the transmitted equivalent. Meanwhile, results from PC-FSSH are close to those from FSSH, but slightly more coherent. However, neither DISH nor PC-FSSH satisfactorily reproduce the flight times of the quantum calculations in the resonance region. Although PC-FSSH does give small oscillations, it is difficult to argue that these correspond to the resonance peaks.

S2-C: Numerical parameters in surface hopping calculations (in atomic units)

For the surface hopping methods, trajectories were started from the initial position $x_0^{cl} = -10$ and ended at $Y_i^{cl} = \pm 10$. The scattering time was then extrapolated to the boundaries x_0 and Y_i for faithful comparison with the quantum results by considering the start and end velocities of the trajectories and free particle motion. For transmission and reflection on the lower surface, this free particle time was calculated as $M\left(|x_0| + |Y_i|\right)/p_i$. For transmission to the upper surface, the free particle flight time was modified slightly, and was given by $M\left(|x_0|/p_i + |Y_i|/\left(p_i - \sqrt{2MA}\right)\right)$ (where A is the potential parameter that determines the upper surface threshold energy). In this way, the trivial free particle motion in the asymptotic region was removed, revealing the effect of the interaction region.

We calculate the flight time for each trajectory, and classify it as either transmission or reflection by whether it ends on the right or the left. We further decompose the total contribution into lower and upper surfaces in proportion to the state-specified population. We also bin the flight times to obtain the time distributions of Fig. 3 in the main text.

The velocity-Verlet algorithm was implemented for nuclear motion, while electronic propagation was performed under the symplectic formalism $\mathbf{c}(t + \Delta t) = e^{-i\mathbf{H}(t)\Delta t}\mathbf{c}(t)$ (Here $\mathbf{c}$ is the electronic amplitude). A time step of $\Delta t = 0.1$ was chosen for all simulations. For DISH, the empirical parameter $w = 2.5$ is used (detailed in Eq. 19 of Ref.[1] and Eq. 24 of Ref.[11]).

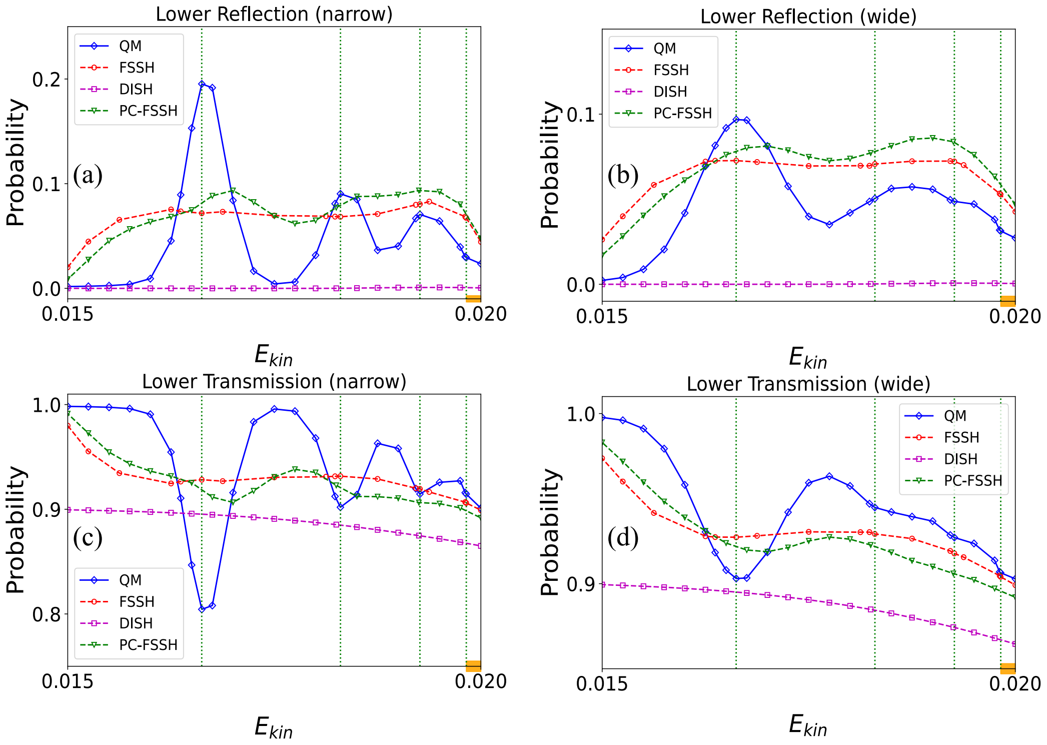

Supporting Figure 1: Energy-dependent scattering probabilities are plotted as functions of the initial kinetic energy for the QM, FSSH, DISH and PC-FSSH results in the resonance region. Panels (a) and (b) show the probability of reflection on the lower surface with the width parameters of the incident Gaussian wavefunction taken as $\alpha = 0.006$ and $\alpha = 0.03$, respectively. Panels (c) and (d) are the same as (a) and (b) but for transmission on the lower surface. In each panel, blue diamonds, red points, magenta squares and green triangles represents results for the QM, FSSH, DISH and PC-FSSH methods respectively (solid or dashed lines with corresponding color are only used to guide the eye). The vertical green short-dashed lines indicate the lowest four (adiabatically corrected) bound energy levels on the upper adiabatic surface.

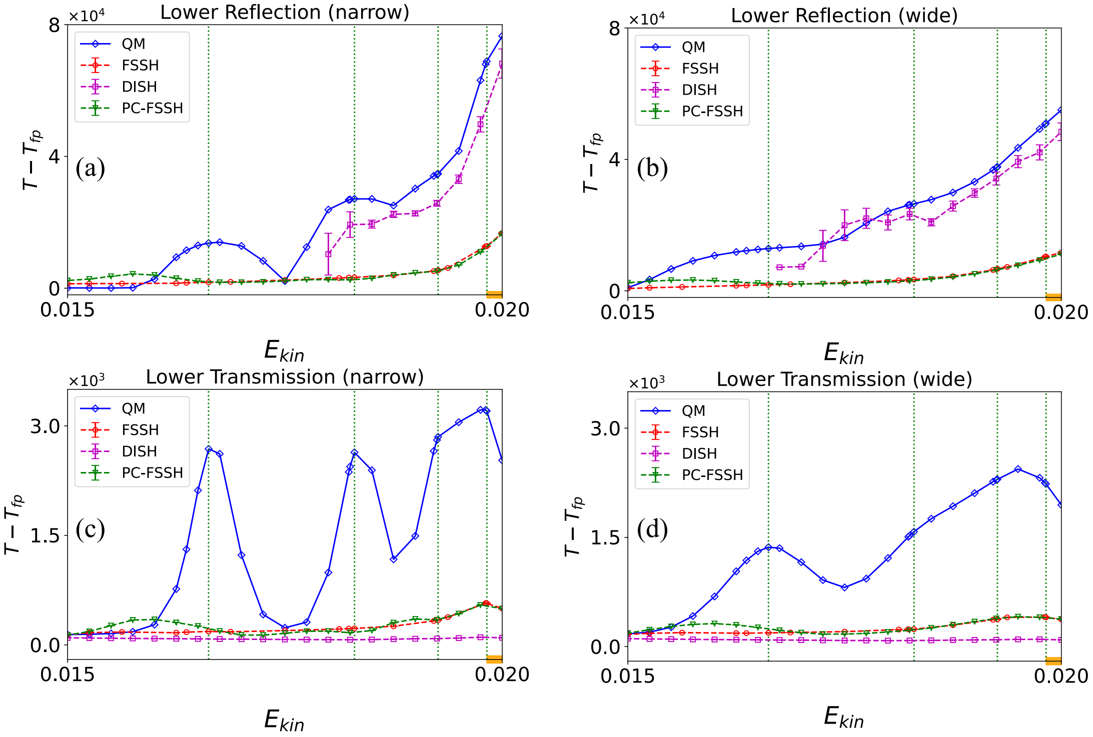

Supporting Figure 2: Mean flight time differences are plotted as functions of the initial kinetic energy for the QM, FSSH, DISH and PC-FSSH results in the resonance region. Panels (a) and (b) show mean flight times for the part of the distribution reflected on the lower surface with the width parameters $\alpha = 0.006$ and $\alpha = 0.03$, respectively. Panels (c) and (d) are the same as (a) and (b) but for transmission on the lower surface. In each panel, blue diamonds, red points, magenta squares and green triangles represent results for the QM, FSSH, DISH and PC-FSSH methods respectively (solid or dashed lines with corresponding color are only used to guide the eye). The vertical green short-dashed lines indicate the lowest four (adiabatically corrected) bound energy levels on the upper adiabatic surface.

S3: The beating phenomenon in the resonance region

In Fig. 3, panel (c2), the QM $\alpha = 0.03$ curve displays strong oscillatory behavior. This is a result of the beating phenomenon due to the influence of the two resonance energies overlapping with the broad-in-energy wave packet. The difference in energy between the two nonadiabatically-corrected energy levels is $E_2 - E_1 = 0.001679$ atomic units. This should correspond to a beating oscillation period of $2\pi/(E_2 - E_1) = 3742$ in atomic units. The time differences between successive peaks were calculated using the split-operator method, and the mean time difference between peaks was found to be less than one percent different from the theoretically-predicted value. For the difference between the uncorrected bound state energies, the oscillation period would be predicted to be 3364 atomic units – which is not the same as the theoretical prediction with the corrections, and not the same as the numerically calculated oscillation period.

S4: The impact of wave packet widths on flight times

In Supporting Fig. 3, we show the mean flight time differences as functions of the initial wave packet width for a number of energies. When only momentum filtering is important,[12–14] the change in the mean time difference would be linearly dependent on the width both in the QM and FSSH cases. Deviation from linearity is observed only for energies in the resonance region in the quantum case (the FSSH nonlinearity in the high-energy region is a numerical artefact of the Monte Carlo averaging). This is another demonstration of the effects of resonant behavior that are not captured by the FSSH method.

Acknowledgement

This work has been graciously supported by a joint grant of the National Natural Science Foundation of China (NSFC) and the Israel Science Foundation (ISF), with NSFC Grant No. 21961142017 and ISF Grant No. 2965/19. We acknowledge the High-Performance

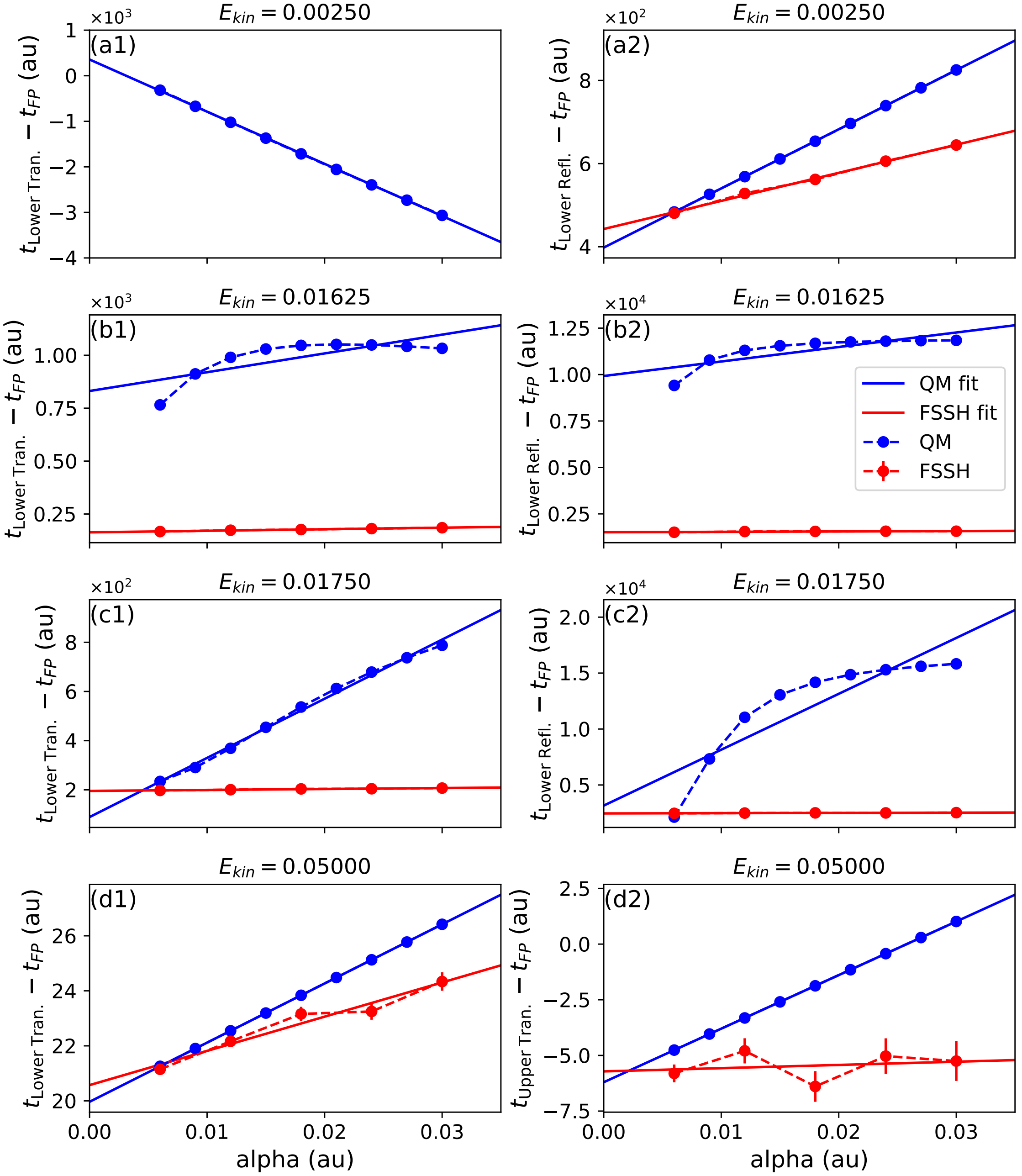

Supporting Figure 3: Mean flight time differences are plotted as functions of the width parameter α for the QM and FSSH results. Panels (a1-a2), show transmission and reflection mean flight time differences on the lower surface with the initial kinetic energy $E_k = 0.00250$. Panels (b1-b2), (c1-c2) are similar to panels (a1-a2) but with initial kinetic energies $E_k = 0.01625$ and $E_k = 0.01750$ respectively. Panels (d1-d2), are similar to (a1-a2) but show transmission on the lower and upper adiabatic surfaces instead, with the kinetic energy $E_k = 0.05000$. Blue points: QM result. Blue lines: linear fitting for QM result. Red points: FSSH result. Red lines: linear fitting for the FSSH result.

Computing Platform of Peking University, Beijng PARATERA Tech CO., Ltd., and the Guangzhou Supercomputer Center for providing computational resources.

References

(1) Jaeger, H. M.; Fischer, S.; Prezhdo, O. V. Decoherence-Induced Surface Hopping. *J. Chem. Phys.* **2012**, *137*, 22A545, doi:10.1063/1.4757100.

(2) Shenvi, N.; Subotnik, J. E.; Yang, W. Phase-Corrected Surface Hopping: Correcting the Phase Evolution of the Electronic Wavefunction. *J. Chem. Phys.* **2011**, *135*, 024101, doi:10.1063/1.3603447.

(3) Dion, C. M.; Hashemloo, A.; Rahali, G. Program for Quantum Wave-Packet Dynamics with Time-Dependent Potentials. *Comput. Phys. Commun.* **2014**, *185*, 407–414, doi:10.1016/j.cpc.2013.09.012.

(4) Colbert, D. T.; Miller, W. H. A Novel Discrete Variable Representation for Quantum Mechanical Reactive Scattering Via the S-Matrix Kohn Method. *J. Chem. Phys.* **1992**, *96*, 1982–1991, doi:10.1063/1.462100.

(5) Yurchenko, S. N.; Lodi, L.; Tennyson, J.; Stolyarov, A. V. Duo: A General Program for Calculating Spectra of Diatomic Molecules. *Comput. Phys. Commun.* **2016**, *202*, 262–275, doi:10.1016/j.cpc.2015.12.021.

(6) Tully, J. C. Molecular Dynamics with Electronic Transitions. *J. Chem. Phys.* **1990**, *93*, 1061–1071, doi:10.1063/1.459170.

(7) Peng, J.; Xie, Y.; Hu, D.; Du, L.; Lan, Z. Treatment of Nonadiabatic Dynamics by On-the-Fly Trajectory Surface Hopping Dynamics. *Acta Phys.-Chim. Sin.* **2019**, *35*, 28–48, doi:10.3866/PKU.WHXB201801042.

(8) Wang, L.; Akimov, A.; Prezhdo, O. V. Recent Progress in Surface Hopping: 2011-2015. *J. Phys. Chem. Lett.* **2016**, *7*, 2100–2112, doi:10.1021/acs.jpclett.6b00710.

(9) Zhu, C.; Jasper, A. W.; Truhlar, D. G. Non-Born–Oppenheimer Trajectories with Self-Consistent Decay of Mixing. *J. Chem. Phys.* **2004**, *120*, 5543–5557, doi:10.1063/1.1648306.

(10) Subotnik, J. E.; Shenvi, N. A New Approach to Decoherence and Momentum Rescaling in the Surface Hopping Algorithm. *J. Chem. Phys.* **2011**, *134*, 024105, doi:10.1063/1.3506779.

(11) Bedard-Hearn, M. J.; Larsen, R. E.; Schwartz, B. J. Mean-Field Dynamics with Stochastic Decoherence (MF-SD): A New Algorithm for Nonadiabatic Mixed Quantum/Classical Molecular-Dynamics Simulations with Nuclear-Induced Decoherence. *J. Chem. Phys.* **2005**, *123*, 234106, doi:10.1063/1.2131056.

(12) Dumont, R. S.; Marchioro II, T. Tunneling-Time Probability Distribution. *Phys. Rev. A.* **1993**, *47*, 85–97, doi:10.1103/PhysRevA.47.85.

(13) Lozovik, Y. E.; Filinov, A. Transmission Times of Wave Packets Tunneling Through Barriers. *J. Exp. Theor. Phys.* **1999**, *88*, 1026–1035, doi:10.1134/1.558886.

(14) Muga, J. G. In *Time in Quantum Mechanics*; Muga, J. G., Mayato, R. S., Egusquiza, Í. L., Eds.; Springer Berlin Heidelberg, **2008**; pp 29–68, doi:10.1007/3-540-45846-8_2.
